# A systematic review of animal and human data comparing the nasal potential difference test between cystic fibrosis and control

**DOI:** 10.1038/s41598-024-60389-9

**Published:** 2024-04-26

**Authors:** Cathalijn H. C. Leenaars, Frans R. Stafleu, Christine Häger, Hendrik Nieraad, André Bleich

**Affiliations:** 1 Institute for Laboratory Animal Science, Hannover Medical School, Hannover, Germany; 2 Department of Animals in Science and Society-Human-Animal Relationship, Utrecht University, Utrecht, The Netherlands

**Keywords:** Animal-to-human translation, Cystic fibrosis, Nasal potential difference, Electrophysiology, Predictive value, Reproducibility, Electrophysiology, Translational research, Animal physiology, Experimental models of disease

## Abstract

The nasal potential difference test (nPD) is an electrophysiological measurement which is altered in patients and animal models with cystic fibrosis (CF). Because protocols and outcomes vary substantially between laboratories, there are concerns over its validity and precision. We performed a systematic literature review (SR) of the nPD to answer the following review questions: A. Is the nasal potential difference similarly affected in CF patients and animal models?”, and B. “Is the nPD in human patients and animal models of CF similarly affected by various changes in the experimental set-up?”. The review protocol was preregistered on PROSPERO (CRD42021236047). We searched PubMed and Embase with comprehensive search strings. Two independent reviewers screened all references for inclusion and extracted all data. Included were studies about CF which described in vivo nPD measurements in separate CF and control groups. Risk of bias was assessed, and three meta-analyses were performed. We included 130 references describing nPD values for CF and control subjects, which confirmed substantial variation in the experimental design and nPD outcome between groups. The meta-analyses showed a clear difference in baseline nPD values between CF and control subjects, both in animals and in humans. However, baseline nPD values were, on average, lower in animal than in human studies. Reporting of experimental details was poor for both animal and human studies, and urgently needs to improve to ensure reproducibility of experiments within and between species.

## Introduction

In its basics, the nasal potential difference test (nPD) is an electrophysiological measurement showing polarisation of the nasal epithelium. In cystic fibrosis (CF), the epithelia are hyperpolarised. Because of the relative ease of measuring potential differences in the nose, and in light of the limitations of the preceding diagnostic technique (i.e. sweat tests), the nPD was further developed as a diagnostic tool, a process which started well before the *CFTR*-gene was discovered. In this process, several laboratories developed standard operating procedures (SOPs). The SOPs were extended to include buffers flowing over the epithelium, which alter the ion flow over the membrane and thereby the potential difference. The use of these buffers, which affect the CFTR and other channels, increased the diagnostic sensitivity of the nPD for CF^1,2^.

Because of the association of nPD with disease severity^3^, the nPD can also be measured in (pre)clinical trials as a proxy of disease severity, both in CF patients and in animal models. Besides, as it reflects the function of the CFTR, it can be used in mechanistic studies. However, while there are standard operating procedures, exact protocols vary between laboratories, and this can in turn affect the measured membrane potential. Consequently, scientists have expressed their concern over the observed variation in protocol and outcomes^4,5^.

The nPD has thus been used for different purposes and in multiple species, and there are valid concerns about its validity and precision^4–7^. Therefore, we performed a systematic literature review (SR) of the nPD. Our SR was designed to answer multiple review questions. In this paper, we describe the relevant data for two of them; first, “is the nasal potential difference similarly affected in CF patients and animal models?”, and second, “is the nPD in human patients and animal models of CF similarly affected by various changes in the experimental set-up?”. This SR of the nPD test follows several narrative reviews of e.g. the nPD in CF patients^8,9^.

Besides being a valuable tool for anyone involved in translational nPD study design, by comparing the same outcome between animal models and CF patients, this SR contributes to the ongoing analysis of translational value of animal studies. Preceding work has shown that the predictive value of animal experiments for humans ranges from 0 to 100%^10^, with relevant differences in average predictive values between medical fields^11^. Straightforward analyses of several potentially relevant factors could not predict the translational value, which means that part of the animal studies that are currently performed cannot be reproduced in humans and thus have limited informative value. While in-depth analyses of combinations of factors may be of future benefit^12^, further in-depth quantitative analyses of specific tests and interventions in animals and humans seem crucial. To the best of our knowledge, our SR is one of the first comparing a specific test; the nPD, between animals and humans.

## Materials and methods

### Protocol

This review followed a protocol that was preregistered on PROSPERO (CRD42021236047) on 05 March 2021. Protocol refinements (i.e. more detailed operationalisations of our planned work) are described in the respective sections below. Except for additional data extraction and analyses, there were no protocol violations. This publication describes the review process (methods), and the data relating to the first and third review question from the protocol, focussing on comparisons between cystic fibrosis and control. Reporting follows the PRISMA guidelines. Other papers will describe the data relating to potential treatment effects (protocol review question 2), the relative sensitivity of the nPD measure compared to other outcome measures in animal models (protocol review question 4), a detailed analysis of study reporting quality, and text frequency analyses of the abstracts of the included studies. This review has also been used as a case study to compare several review tools (manuscript under review).

### Search

PubMed and Embase (via Ovid) were searched on 23 March 2021, without restrictions for publication date or language. Separate search strings were used for: animals and humans, CF, and the nPD. Strings comprised both title-abstract-keyword terms as well as thesaurus (MeSH/Emtree) terms. The comprehensive animal filters, mentioning all potentially-relevant species in single and plural as < [tiab] > or < .ti,ab,kw. > terms, as well as all relevant thesaurus terms in the appropriate syntax, have been published elsewhere^13^. The other search stings are provided in Table 1.

**Table 1:** Search strategy.

Pubmed	
nPD	Membrane potentials [MeSH:NoExp] OR ((Nasa*[tiab] OR naso*[tiab] OR membran*[tiab] OR transmembran*[tiab]) AND (Potential[tiab] OR potentials[tiab] OR voltage[tiab] OR voltages[tiab] OR current[tiab] OR currents[tiab]) AND (Difference[tiab] OR differences[tiab] or change[tiab] OR changes[tiab] OR alteration[tiab] OR alterations[tiab] OR variance[tiab]))
CF	Cystic fibrosis [MeSH] OR Mice, Inbred CFTR [MeSH] OR Cystic Fibrosis Transmembrane Conductance Regulator [MeSH] OR (cystic[tiab] AND (fibrosis[tiab] OR fibroses[tiab] OR fibrotic[tiab])) OR Mucoviscidos* [tiab] OR Mucoviscoid* [tiab] OR Mukoviszid* [tiab] OR CFTR [tiab] OR Fibrocystic Disease [tiab] OR Fibrocystic Diseases [tiab] OR Mckusick [tiab] OR CFRD [tiab] OR "pancreas cystic disease" [tiab] OR muco-patient* [tiab] OR muko-patient* [tiab] OR (CF [tiab] AND (lung [tiab] OR lungs [tiab] OR pulmonary [tiab] OR ABPA [tiab] OR mucus [tiab] OR liver [tiab] OR livers [tiab] OR steatosis [tiab] OR cirrhosis [tiab] OR cirrhotic [tiab] OR meconium ileus[tiab] OR gastrointestinal [tiab] OR intestine [tiab] OR intestines [tiab] OR intestinal [tiab] OR duodenum [tiab] OR jejunum [tiab] OR colon [tiab] OR caecum [tiab] OR DIOS [tiab] OR ((sweat [tiab] OR eccrine [tiab] OR apocrine [tiab] OR salivary [tiab] OR parotid [tiab] OR sublingual [tiab] OR submandibular [tiab] OR sub-lingual [tiab] OR sub-mandibular [tiab] OR von Ebner [tiab]) AND (gland [tiab] OR glands [tiab])) OR ((Paranasal [tiab] OR Para-nasal [tiab] OR frontal [tiab] OR ethmoidal [tiab] OR maxillary [tiab] OR sphenoidal [tiab]) AND (sinus [tiab] OR sinuses [tiab])) OR pancreas [tiab] OR pancreatic [tiab]))
Human	clinical study [pt] OR clinical trial [tiab] OR intervention study [tiab] OR “clinical studies as topic”[MeSH] OR first in man [tiab] OR proof of concept [tiab] OR randomized [tiab] OR placebo [tiab] OR drug therapy [sh] OR randomly [tiab] OR trial [tiab] OR groups [tiab] OR multicenter study[pt] OR “Multicenter Studies as Topic” [Mesh]
Embase	
nPD	exp potential difference/OR (exp nose/AND exp electrical parameters/) OR ((Nasa* OR naso* OR membran* or transmembran*) AND (Potential OR potentials OR voltage OR voltages OR current OR currents) AND (Difference OR differences or change OR changes OR alteration OR alterations OR variance)).ti,ab,kw
CF	Cystic fibrosis/OR cystic fibrosis transmembrane conductance regulator/OR (cystic adj2 fibros*).ti,ab,kw. OR fibrocystic diseas*.ti,ab,kw. OR (mucovisc* or Mukoviszidose).ti,ab,kw. OR CFRD.ti,ab,kw. OR muco-patient*.ti,ab,kw. OR muko-patient*.ti,ab,kw. OR pancreas cystic disease.ti,ab,kw. OR pancreas fibrocystic disease.ti,ab,kw. OR pancreas fibrosis.ti,ab,kw. OR pancreatic cystic disease.ti,ab,kw. OR pancreatic fibrosis.ti,ab,kw. OR (CF adj30 (lung OR liver OR stomach OR intestines OR pulmonary OR meconeum ileus OR gastrointestinal OR intestine OR intestines OR intestinal OR pancreas OR pancreatic OR ((sweat OR eccrine OR apocrine OR salivary OR parotid OR sublingual OR submandibular OR von Ebner) adj2 (gland OR glands)) OR ((Paranasal OR frontal OR ethmoidal OR maxillary OR sphenoidal) adj2 (sinus OR sinusses)))).ti,ab,kw
Human	exp clinical trial/OR clinical study/OR human subject.ti,ab,kw. OR clinical drug trial.ti,ab,kw. OR major clinical trial.ti,ab,kw. OR trial, clinical.ti,ab,kw. OR clinical study.ti,ab,kw. OR phase 1 clinical trial.ti,ab,kw. OR phase 2 clinical trial.ti,ab,kw. OR phase 3 clinical trial.ti,ab,kw. OR clinical trial, controlled.ti,ab,kw. OR clinical trial, phase 1.ti,ab,kw. OR clinical trial, phase 2.ti,ab,kw. OR clinical trial, phase 3.ti,ab,kw. OR clinical trials.ti,ab,kw. OR clinical trial, phase I.ti,ab,kw. OR clinical trial, phase II.ti,ab,kw. OR clinical trial, phase III.ti,ab,kw. OR intervention study.ti,ab,kw

The reference lists of relevant reviews and included studies were screened by two independent reviewers (FS & CL) to retrieve additional eligible studies.

### Screening

Screening was performed in two distinct phases: title-abstract screening and full text screening, following the criteria in Table 2. Screening was performed by two independent reviewers (FS & CL) for both phases, in two separate projects in Rayyan^14^. Discrepancies could all be resolved by discussions between the reviewers.

**Table 2:** Exclusion criteria.

Exclusion criteria	Screening phase	
	Title-abstract	Full text
Study not about CF	X	X
Study not in vivo^a^	X	X
No nPD measured	X	X
No untreated control group (either with or without CF)^b^ present		X
No full peer-reviewed publication^c^		X

There were no restrictions for publication date or language.

^a^Ex vivo, in vitro and in silico models were excluded.

^b^Included were both studies that compared CF with healthy controls at baseline, and studies that compared treated with untreated CF.

^c^Conference proceedings and short communications lacking a detailed description of the methods were excluded because we planned analyses of the experimental set-ups.

### Data extraction

Data were extracted by two independent reviewers (FS & HN or FS & CL) in Covidence^15^, in two distinct phases. In the first phase, basic data on study design and included population were extracted. This allowed us to categorise the included studies into the CF versus control comparisons included in this publication, and the treatment studies to be published at a later stage. In the second phase, all data presented in this publication were extracted for the relevant subset. Discrepancies were resolved by discussion between the reviewers. When standard deviations (SDs) were provided, they were converted to Standard Errors (SEs). When values were not provided in text or tables, digital rulers were used.

It became clear from the first data extractions that many alternative nPD measures are reported. The nowadays common low chloride nPD values are less sensitive to infection and mild trauma, and more stable and reliable in distinguishing CF from non-CF^1^. However, many of the older studies only report baseline measures. To include data from as many studies as possible, we decided to separately extract both baseline and low chloride measurements.

Baseline measures were preferentially extracted without flow or with only buffer, but if these data were not available, measurements in the presence of amiloride were extracted. Low or zero chloride data were preferentially extracted in combination with the additional stimulation by forskolin or isoproterenol, but when data with these chloride channel activators were lacking, low/no chloride buffer data without these activators were extracted.

When a paper described multiple comparisons, we only included one of these comparisons to prevent dependency of data. We preferentially included the first one performed, or when a cross-over design was used, the first one presented. For example, De Wachter et al.^16^ compared two different nPD methods in CF and control subjects. We included the first described into our analyses; the one with the subcutaneous agar-filled needle as the reference electrode. Similarly, for Duperrex et al.^17^, values from the inferior turbinate were included. We only deviated from this method when a later-presented sample was substantially larger, as e.g. the third protocol described in^1^.

When repeated baseline measures of the same condition were performed in the same session, we took the last measurement of this condition. When repeated treatment effects were separately presented, we selected the last one within the “treatment” period.

### Risk of bias assessments

Risk of bias (RoB) was assessed independently by 2 reviewers using a list of questions based on the SYRCLE RoB tool^18^ and the Cochrane Collaboration’s tool^19^. Besides, compliance with all items from the ARRIVE^20^ and CONSORT^21^ guidelines was assessed as a reflection of reporting quality. All discrepancies were resolved by discussion between these reviewers, the third reviewer specified in the protocol was not necessary. In this paper, we summarise the RoB for the papers included in these CF versus control analyses following the SYRCLE and Cochrane tools. RoB for treatment effects has been published with those findings^22^.

The SYRCLE and Cochrane RoB tools consists of separate domains for studies included in systematic reviews. The tools are both focussed on the comparison of treatment versus control. This makes them perfectly applicable for the data we described in our parallel publication of this SR, but less so for the CF-control comparisons described in this one. As the CF or control genotype is present from birth, the risk of bias associated with treatment allocation is irrelevant. For this publication we restrict RoB reporting to the relevant domains, as summarised in Table 3. Findings will be presented in summarised form per domain and per study. RoB and reporting quality will be published in more detail at a later stage.

**Table 3:** Analysed risk of bias domains (based on^18^ and^23^) described in this publication.

Bias domain	Type of bias	Operationalisation^a^
Baseline characteristics	Selection bias	Where the CF and control subjects comparable at baseline, at least for age and sex?
Housing	Performance bias	Was housing of animals mixed over the cages or adequately randomised/counterbalanced?
Blinding	Performance bias	Were all involved in contact with the subjects adequately blinded?
Outcome assessment	Detection bias	Was the order of measuring CF and control subjects adequately randomised/counterbalanced?
Blinding	Detection bias	Were those performing the nPD and those analysing the traces adequately blinded?
Missing outcome data	Attrition bias	Were missing outcome data transparently reported and equally distributed over CF and control groups?
Selective outcome reporting (“cherry picking”)	Reporting bias	Was a protocol posted before study start, and were all planned outcomes reported?
Other	Other bias	Were there no other reasons for concerns of biassing the results?

^a^Yes reflects a low risk of bias, no reflects a high risk of bias, unclear reflects insufficient information to be sure.

### Analysis

Data were exported from Covidence to Excel. Data were checked and cleaned in Excel. Cleaning comprised manual reintroduction of vanished decimal separators (due to language settings that were not fully compatible with Covidence export), conversion of all flow rates to mL/min, splitting of ranges for age and weight to minimum and maximum values, selecting median (outcomes) or minimum (n) values for reported ranges, calculating the median for other measures occasionally reported as a range, estimating SDs from InterQuartile Ranges (IQRs, four values from one study, using the formula from the Cochrane handbook^23^: [SD ≈ (q3 − q1)/1.35]), ranges (26 values from 8 studies, using the formulas suggested for the respective sample size from Hozo et al.^24^ and Confidence Intervals (CIs, two values from one study, using the formula from the Cochrane handbook^23^: [SE = (upper limit – lower limit)/3.92] and converting them to SEs, adding columns for categorised variables and risk of bias counts per study, and adapting variable names, text and factor variables to be compatible with R.

All analyses were performed in R^25^ via RStudio, using the following packages: readxl^26^, dplyr^27^, ggplot2^28^, tm^29^, tidyr^30^, crosstable^31^, meta^32^ and metafor^33^. Two papers described both animal and human data^4,34^. Because we performed all analyses at the paper level, these two papers were excluded from all analyses other than the bibliographic ones. Comparisons of fractions of animal and human papers were made with Wilcoxon’s rank sum test.

Some authors report the potential as is (i.e., negative at baseline, e.g.,^35^). Others plot negative values in the positive [e.g.^36^], or reverse the potential in reporting [e.g.^37^]. Consequently, for baseline potential differences, we analysed absolute values. This should result in meaningful analyses with interpretable outcomes. We calculated a simple unweighted Spearman’s correlation coefficient for absolute within-study nPD values in CF versus control, using base R, prior to our planned analyses.

Responses to low or zero chloride solutions were reported as absolute values with and without reversion, but also as absolute and percent changes compared to baseline. As these values can cross zero, analysing absolute values would not result in meaningful analyses with interpretable outcomes. Thus, we analysed absolute difference scores between the CF and the control values. As these data were extracted in addition to the protocol, we did not design these analyses upfront.

The following weighted meta-analyses were performed:

1. A planned overall translational meta-analysis for the baseline difference in nPD between cystic fibrosis and control without treatment, comparing human patients and animal models as subgroups. This analysis used standardised mean differences and the “metacont” function from the meta package^32^.
2. an additional (unplanned) overall translational meta-analysis for the difference in nPD between cystic fibrosis and control after low or no chloride buffer perfusion, comparing human patients and animal models as subgroups. This analysis used the “metagen” function from the meta package^32^.
3. a planned meta-regression analysis assessing the effects of buffer perfusion speed and species (mouse or human) on the baseline nPD. This analysis used the “rma” function from the metafor package ref^33^. As a rule of thumb, regression analyses should not be performed if there are fewer than 10 observations for a specific (level of a factorial) variable. Therefore, our protocol specified restriction of meta-regression to variables for which we had data from at least 10 papers. In line with this idea, but not explicitly described in our protocol, we excluded studies of pigs (k = 2 included papers) and rats (k = 4 included papers) from this meta-regression.

All meta-analyses used a frequentist (classical) random effects model with standardised mean differences. Heterogeneity between the studies was assessed using the I^2^ statistic. Publication bias was assessed with funnel plots and trim&fill analyses.

## Results

### Study flow and included literature sample

**Figure 1:**
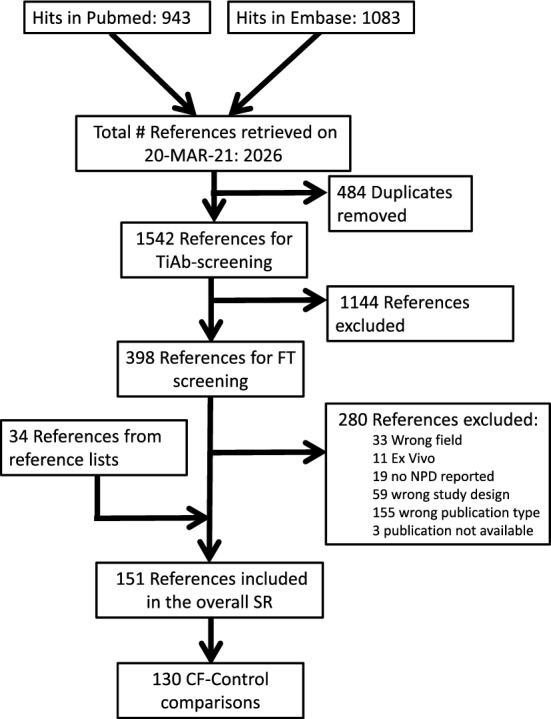
Reference flow.

The flow of studies from search to included is summarised in Fig. 1.

The 130 papers describing baseline CF-control comparisons comprise 55 papers about animal studies, 73 about human studies, and 2 describing both. Of the 55 animal studies, 13 reported multiple relevant experimental groups. Of the 73 human studies, 16 reported multiple relevant experimental groups. The number of relevant CF-control comparisons per paper was 2 for 28 papers (12 animal papers, 14 human papers and 2 papers describing both an animal and a human CF-control comparison), and 3 for the remaining 3. In animal SRs, it is common practice to extract data from different experiments within a single reference, and even from different groups analysed within a single experiment, and analyse them as if the data were independent. Following that practise, we could in total have included 164 (99 + 28 × 2 + 3 × 3) comparisons. However, particularly for this baseline comparison, we were worried about issues with correlated data. To prevent these, data were only extracted and analysed for the first-described experimental CF-control comparison meeting the inclusion criteria.

**Figure 2:**
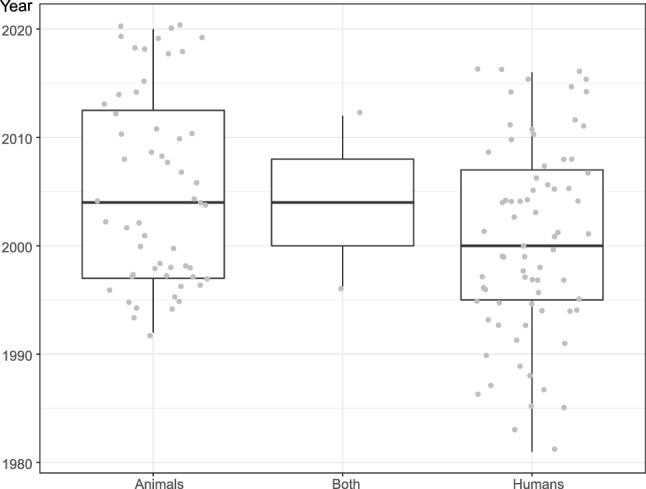
Publication dates by study population.

The included nPD animal studies were published from 1992 through 2020; the human studies from 1981 through 2016 (Fig. 2). Animal studies were on average published more recently than human studies (Wilcoxon rank sum test: W = 2540, p = 0.01).

The 4 most popular journals were the American journal of respiratory and critical care medicine (k = 2 animal and k = 12 human papers); the American journal of respiratory cell and molecular biology (k = 4 animal and k = 3 human papers); Human gene therapy (k = 4 animal and k = 2 human papers); and Thorax (k = 1 animal and k = 5 human papers).

The language of most papers (k = 126, including all animal papers) was English. Three human papers were in French, 1 was in German. We extracted the countries in which the protocols for the included studies had been submitted to ethical review. These data were available for k = 81 of the 130 included papers. The majority of the studies was approved in the USA (k = 18 animal and k = 16 human studies). Other countries with more than 5 approved studies were Belgium (overall k = 7), France (k = 7), Germany (k = 6) and Canada (k = 6).

A simple text frequency analysis of the titles of the included studies showed no relevant differences in the main topics covered between included animal and human studies (data not shown).

### Population

**Figure 3:**
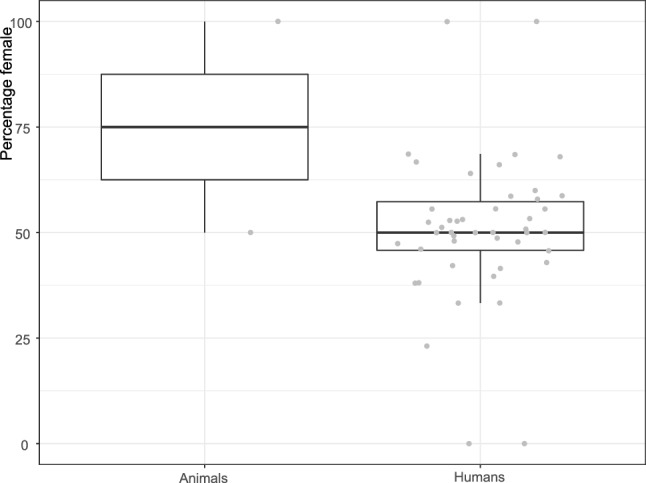
Percentage female by species.

Because we performed all analyses at the paper level, the two papers describing both animal and human studies^5,34^ were excluded from further analyses. The animal species studied were mice (k = 49), rats (k = 4) and pigs (k = 2). Sex/gender was reported in k = 68 (53.1%) of the included papers. Most of these analysed both males and females (k = 63), 2 exclusively analysed males, 3 exclusively analysed females. Animal papers were responsible for the main part of underreporting, only two out of the 55 animal papers reported sex. In the studies of both genders /sexes, the percentage female varied from 11.8 to 89.0 (Fig. 3), with median values for CF and control groups around 50%.

The most studied mutation was the p.F508del mutation (k = 22 animal and k = 9 human papers). Multiple mutations were analysed in 2 (out of 55) animal and 22 (out of 73) human papers. The next commonly studied mutation was the *CFTR* knock-out (k = 23 animal papers). Other mutations (e.g. G551D and G542X) were specifically studied in fewer than 5 of the included papers. Information about the analysed mutation(s) was not reported in 1 animal and 39 human papers. While many other CF animal models exist^38^, all included animal studies described genetic animal models. Disease severity was (briefly) mentioned in 30 human and 2 animal papers. In humans, it varied from “not severe” to “frequently hospitalised”. In animals, one mentioned that the disease was not severe^39^, another described strain differences for CF disease severity^36^.

Some information on age or weight of the subjects was reported in 27 (out of 55) animal and 60 (out of 73) human studies. Animal (mouse) ages ranged from 0 to 56 weeks, human ages from 0 to 70 years. Mouse weight varied from 15 to 53g. The immune status was only reported in 7 animal papers, all specific pathogen-free. Prevention of intestinal obstruction (a common problem in CF animal models) was described in 25 animal papers and targeted diet (k = 2 liquid, k = 1 other), laxatives (k = 1), a combination of diet and laxative (k = 1) and genetic correction of the gut (k = 2).

Of the human papers, 16 mentioned something about ongoing background treatments (e.g. “no regular medications” or “pancreatic supplements and antibiotic treatment”). None of the animal papers described co-medication.

### Experimental design

None of the papers included in this part of the review reported protocol registration. Note that registered protocols were more common in the treatment vs. control comparisons.

Part of the methods were referenced by 33 (out of 55) animal and 53 (out of 73) human papers, explaining part of the underreporting of experimental details described below. However, several authors only half referenced the methods (e.g. “a modification of …” or “adapted from …”^36,40^), without details on what was modified and adapted, which limits reproducibility.

**Figure 4:**
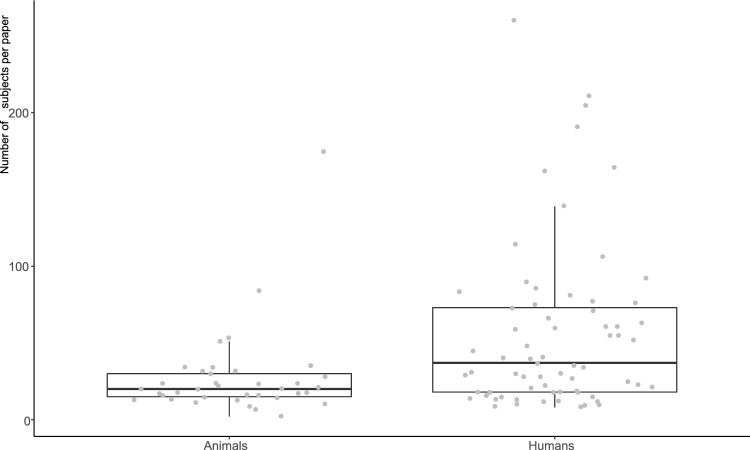
Total number of subjects for which data were extracted per paper.

Total numbers of study subjects were reported by 41 (out of 55) animal and 70 (out of 73) human papers, and ranged from 2 to 175 for animal and from 8 to 260 for human papers. On average, human studies were significantly larger than animal studies (W = 784, p = 0.001, Fig. 4).

CF animals were mainly compared to heterozygous animals (k = 32). The use of control littermates (wildtype, heterozygous or both) were only specified in k = 9 papers. CF patients were mostly compared to healthy controls (k = 63), with or without the exclusion of specific diseases. Repeated nPD measurements were reported in k = 5 animal papers and in k = 15 human papers. Animals were measured up to 5 times, humans up to 6 times. Time between repeated measurements varied from a day to nearly 2 months.

Only two papers provided information on the time the tests were performed; k = 1 animal study described performing the tests “throughout the day”^35^, one human paper “in the afternoon”^41^. For studies of nocturnal rodents, the light cycle can be reversed. Only k = 5 (out of 55) animal studies provided some information about the light cycle, which was normal (k = 2), 12:12 (k = 2) and reversed (k = 1). Only 3 animal (out of 128) papers reported laboratory temperature, which was 21 °C (k = 2) or 22 °C (k = 1). None of the included papers reported laboratory humidity.

The number of subjects leaving the study before the last nPD (“DropOuts”) were only mentioned by two human papers (which had none, or at least 4).

### nPD technique

For animals, the nPD can only be performed under anaesthesia. The anaesthetics used were reported in k = 33 (out of 55) animal papers. The most commonly used combination anaesthetic was ketamine-xylazine. Other combinations and single anaesthetics were used in fewer than 3 papers each.

Information about the measuring electrode was reported in 32 (out of 55) animal and 51 (out of 73) human papers. Commonly used measuring electrodes were various Foley catheters, (pulled) PE-10 and PE-20 tubing, umbilical catheters and double-lumen silicone rubber tubes. Information about the bio-electrical connection was reported in 19 (out of 55) animal and 45 (out of 73) human papers and comprised continuous flow of the baseline buffer, agar bridges or electrode cream.

Information about the placement of the measuring electrode was provided in k = 37 (out of 55) animal and k = 58 (out of 73) human papers. For the animal papers, this information was mainly “in a nostril”, with or without a depth. For human papers, terms used in more than 5 papers were “under the inferior turbinate” and “floor of the nasal cavity/nose”. Testing of the placement was described for k = 5 animal and k = 29 human papers. Most of this placement testing comprised advancement or withdrawal of the measuring electrode up to the maximal potential difference.

Information about the placement of the reference electrode was provided in k = 29 (out of 55) animal and k = 52 (out of 73) human papers. In humans, the reference electrode was most commonly placed in (subcutaneously) or on (abraded skin) the forearm. In animals, it was mainly placed subcutaneously. If the location was specified, it was mostly the abdomen or a paw.

The perfusion solutions were reported for k = 51 (out of 55) animal and 57 (out of 73) human papers. Most frequently used were various solutions with Amiloride, different buffers with no or low chloride, and different types of Ringer. The only noticeable difference between animal and human studies was the use of Isoproterenol, which was more common in animal studies (k = 29 versus k = 6). Duration of the perfusion was reported for k = 32 (out of 55) animal and k = 35 (out of 73) human papers. It ranged from 0.2 to 47.5 min for animals, and from 1.2 to 35.0 min for human subjects. Information about the perfusion device was reported in 16 (out of 55) animal and 15 (out of 73) human papers, and comprised various types of peristaltic, microperfusion and syringe pumps.

Information about the baseline buffer composition was reported in 47 (out of 55) animal and 52 (out of 73) human papers. It comprised exact concentrations, but standard (HEPES-buffered) Ringers and Krebs solutions were also regularly reported. The perfusion speed was reported in 32 (out of 55) animal and 39 (out of 73) human papers. The mostly used units to describe the flow were mL/min (k = 2 animal and k = 32 human papers), µL/min (k = 15 animal and k = 2 human papers) and mL/h (k = 9 animal and k = 3 human papers). After conversion to mL/min, flow ranged from 0.00001 to 0.1 for mice, and from 0.05 to 5.0 for humans.

### nPD outcomes in CF and control

Overall, values were estimated (from a provided graph/range/CI/IQR) for at least one of the outcomes (baseline or low chloride nPD) or their associated parameters (SEs or n) for 48 out of 128 papers. Because baseline nPD values are all negative, but some authors reverse the sign, we analysed absolute values for baseline values and difference scores for low chloride nPDs.

**Figure 5:**
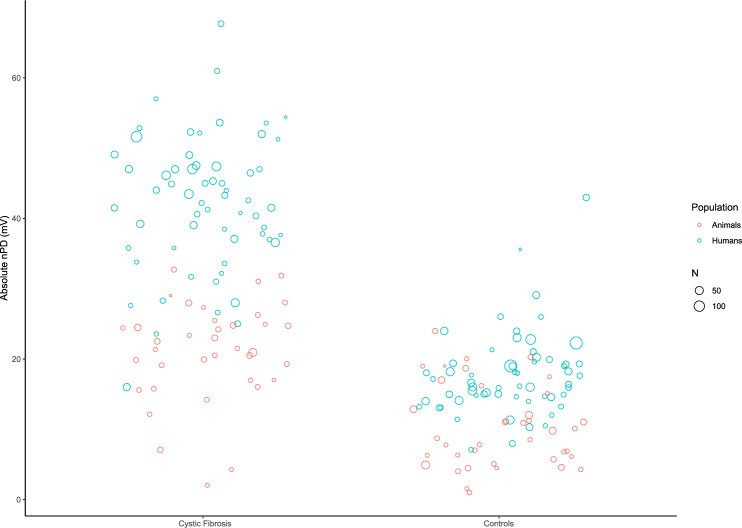
Baseline nPD values in animals and humans with and without CF.

Baseline nPD data were available for 43 (out of 55) animal and 62 (out of 73) human papers. For animals, absolute control nPDs ranged from 1.0 to 24.0 mV, with a median value of 9.0mV, and absolute CF nPDs from 2.0 to 32.7mV, with a median value of 21.5mV. For humans, absolute control nPDs ranged from 7.1 to 43.0mV, with a median value of 16.9mV, and absolute CF nPDs from 16.0 to 67.7mV, with a median value of 43.0 (Fig. 5).

**Figure 6:**
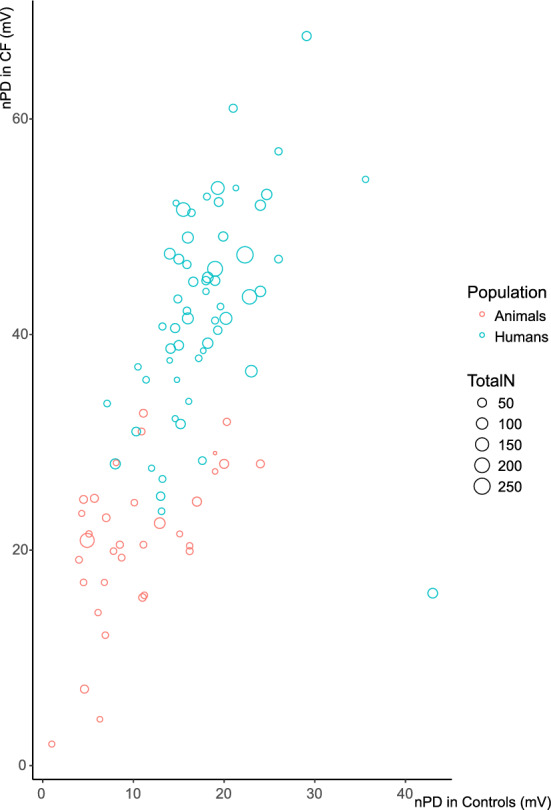
Correlation of baseline CF and control nPD values.

A clear within-study correlation between CF and control values (Fig. 6, r(103) = 0.69, p < 0.01, uncorrected for study size) suggests that while there is a convincing and reproducible difference in the nPD values between CF and control (Fig. 5), the values heavily depend on the local protocols, and the animal protocols on average result in lower values than the human protocols.

**Figure 7:**
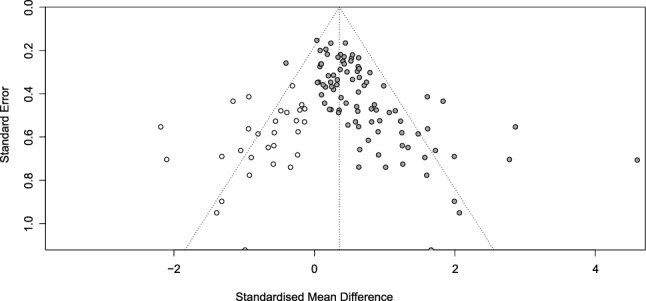
Funnel plot. Open circles reflect added studies.

A meta-analysis of the nPD in CF versus control, subgrouped by animal vs. human studies, showed a clear overall difference between CF and control (Overall SMD: 0.56; 95%-CI: 0.46/0.66; z = − 10.79; p < 0.0001), and a trend-level difference between animals and humans (Q = 3.48; df = 1; p = 0.062). However, heterogeneity was high (I^2^ = 50.4% overall, 63.1% for animals and 38.5% for humans) and small study effects were observed. In the trim-and-fill analysis, 30 studies were added, and the overall difference between CF and control became smaller (Overall SMD: 0.36; 95%-CI: 0.21/0.50; z = 4.89; p < 0.0001, Fig. 7).

Low chloride nPD data were available for 38 (out of 55) animal and 47 (out of 73) human papers. For animals, absolute CF-control difference scores ranged from 0.7 to 24.8 mV, with a median value of 10.9 mV. For humans, difference scores ranged from 1.6 to 36.4 mV, with a median value of 16.0mV. The meta-analysis shows an overall difference between CF and control (z = 16.6; P < 0.0001), and between the animal and human subgroups (Q = 16.1; p < 0.0001). Note that heterogeneity was high; I^2^ = 95.4% (95% CI 94.7–96.0%). Besides, small study effects were present. In the trim and fill analysis 19 studies were added and the overall difference between CF and control became smaller.

### Meta-regression

The only relevant variables with sufficient data (k ≥ 10; k ≥ 10 per category for categorical variables) to analyse in the planned meta-regression were buffer flow rate and species. Note that while data from rats (k = 4) and pigs (k = 2) were included in all analyses described above, they were excluded from the meta-analyses. The results from the meta-regression are shown in table 4.

**Table 4:** Results from the meta-regression.

Variable	Range/values	Estimate (SE) in mV	p-value
(Intercept)		9.73 (0.90)	< 0.0001
Buffer flow rate (mL/min)	0.00001 to 5.0	0.38 (0.40)	0.35
Species	Mice/humans	8.26 (1.64)	< 0.0001

### RoB

**Figure 8:**
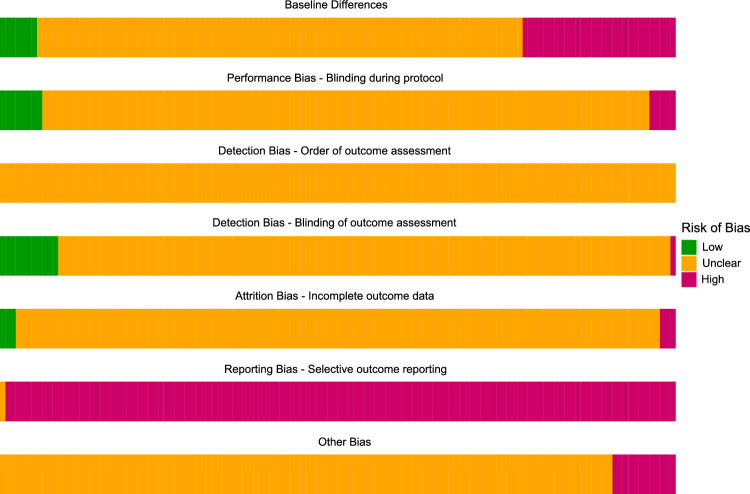
Summary of the risk of bias.

The median number of RoB items scored “unclear” per study was 7 for animal and 5 for human papers, and the number ranged from 2 to 7. Human studies scored significantly fewer “unclears”, reflecting more complete reporting, than animal studies (W = 3667, p < 0.001). The median number of RoB items scored “high” per study was 1 for both animal and human papers. The median number of RoB items scored “low” per study was 0 for both animal and human papers. The scores of the included studies per item are shown in Fig. 8, except for the item “housing”, which is unique to animal studies. Risk of bias due to non-random or unbalanced housing was unclear for all k = 55 included animal studies. More detailed presentation of our analyses of RoB and reporting quality will be presented in a separate publication.

## Conclusions

This SR summarises 130 papers comparing baseline nPD values between CF and control. It confirmed substantial variation in the experimental design between groups, as previously described^42^. These variations may contribute to differences in the nPD outcome value. Consistent with the observation that the human nPD is a reproducible test when performed in a single centre, and extending this observation to animal studies, we showed a clear correlation between CF and control values within studies. Our study further confirmed a clear difference in baseline nPD values between CF and control, both in animals and in humans. However, baseline nPD values were, on average, lower in animal than in human studies.

To the best of our knowledge, this is the first SR of the nPD, and one of the few SRs directly comparing a specific outcome between animals and humans. Other systematic animal-human comparisons focussed on dental outcomes^43–45^ and haemodynamic effects (blood pressure, heart rate)^46^. Experimental design was also previously compared between animal and human studies in a systematic manner^47^. We here provide a full overview of the use of the nPD up to our search date (23 March 2021), which showed relevant differences between animal and human studies.

While this search date could be seen as a limitation, SRs generally take a long time to complete, with a median of 66 weeks and extremes up to 186 from start to completion^48^. Full review updates are crucial if the newly available literature could alter the conclusions of a review. For the here-presented baseline comparisons of CF versus control in animals and humans we would not expect new data to change the conclusions, but additional data could potentially allow us to perform more meaningful analyses of factors affecting the nPD. Thus, we tested if an update would be informative, by a brief analysis of an updated PubMed search performed on 26 May 2023, as described in the discussion of our preceding manuscript of treatment effects^22^. Only five new references met our inclusion criteria for the here-presented CF-control comparisons^49–53^. Based on this small number, particularly with the observed low level of reporting experimental details (discussed below), we do not expect a full update to allow for more meaningful analyses. A formal update would delay publication of these findings further, especially with peer review currently lasting over 6 months. Thus, we do not think an update is currently warranted.

In general, reproducibility of scientific experiments depends on knowledge of the experimental design details. For this SR, reporting of experimental details was worse than anything we came across before (the authors have worked on over 20 other SRs). Variables of specific concern are disease severity (only reported in 32 papers), background treatments (only reported in 16 human papers), time of testing (only reported in very broad terms in two papers) and the number of subjects not completing the experiments (only reported in 4 papers). Besides, outcome measures varied between studies. Furthermore, specific to animal studies, details about husbandry (light cycle) and the tested sexes were frequently lacking. The poor reporting hinders reproducibility, but it also limited the analyses we could perform. To ensure reproducibility of experiments within and between laboratories and species, we strongly encourage everyone reporting nPD experiments to not only follow the arrive guidelines, but also report in full all details about the used voltmeters, electrodes and other equipment used.

Concerning data synthesis, the limited reporting of experimental details prevented us to include additional variables in our meta-regression. In the other part of this review, where we were planning to synthesise treatment effects, the limited reporting even prevented any relevant analysis from being performed^22^. We hope that reporting of experimental detail will improve over time, which would allow for more meaningful analyses in future systematic reviews.

Besides limitations due to poor reporting, five further limitations restrict the reliability of our summary findings. First, several of the included papers describe datasets that overlap to some extent, second, our search may have missed relevant references, third, the results may be confounded, fourth, small study effects were present, and fifth, risk of bias in the included studies was substantial.

We tried to prevent overlap in included data sets by excluding data that fully overlapped; the previously reported control data from^54^, which fully overlap with two other included publications^55,56^, were thus excluded from all analyses. With partial overlap we would have lost information by excluding summary values, and we could not always be fully aware of the (extent of the) overlap anyhow. There is one example where overlap is certainly relevant: four of the pigs described in Chen et al. 2010^57^ were also part of the analyses by Rogers et al.^58^.

The sensitivity of our search may be disputed, because “snowballing” retrieved 34 additional references. An informal analysis of the (indexing) terms used in these references shows that several of the additional references did not mention the nasal potential difference in title, abstract, or author-provided keywords, and that the databases did not add index terms for potential differences for these records either. However, most added references were missed by our searches because our human search string was developed to retrieve translational studies, focussing on randomised clinical trials. For future literature studies it needs to be extended; terms reflecting different human study types (case–control studies and diagnostic studies for example) need to be added.

The animal-human comparison could be confounded by publication date, as we found a clear difference between the mean publication date of the animal and human studies. Besides, we cannot rule out confounding for variables which were poorly reported, for example sex and time of testing. While most of these variables theoretically do not affect the nPD, caution with interpretation of the findings in this review is warranted.

For future literature analyses on nPD, we recommend extended data extraction to allow for further analyses of the experimental set-ups, e.g. adding the warming of the perfusion solutions^59^, used voltmeters, used electrodes, and tilt for rodent studies. These were not formally analysed for this SR, but our casual observations during extraction suggests that these data were not reported less than the ones we analysed.

In this review, we found that nPD outcome effect sizes are not fully comparable between animals and humans. However, the nPD and the CF animal models show resemblance with human CF patients, indicating construct validity of the animal models. To ensure reproducibility of experiments within and between species, there is a huge need to improve reporting.

## Data Availability

All used search strings are publicly available, either within this this manuscript or in one of the references. All search results, screening results and extracted data are available on OSF (https://osf.io/fkyhc/).

## Abbreviations

CF: Cystic fibrosis
CFTR: Cystic fibrosis transmembrane conductance regulator
CI: Confidence interval
HEPES: 4-(2-Hydroxyethyl)-1-piperazineethanesulfonic acid
IQR: Interquartile range
nPD: Nasal potential difference
RoB: Risk of bias
SD: Standard deviation
SE: Standard error
SOP: Standard operating procedure
SR: Systematic literature review
